# Severe skin reaction secondary to concomitant radiotherapy plus cetuximab

**DOI:** 10.1186/1748-717X-3-5

**Published:** 2008-01-28

**Authors:** Bernhard Berger, Claus Belka

**Affiliations:** 1 Department of Radiation Oncology, University of Tübingen, Hoppe-Seyler-Str. 3, 72076 Tübingen, Germany

## Abstract

The therapeutic use of monoclonal antibodies against the epidermal growth factor receptor (EGFR) is specifically associated with dermatologic reactions of variable severity. Recent evidence suggests superiority of the EGFR inhibitor (EGFRI) cetuximab plus radiotherapy compared to radiotherapy alone in patients with squamous cell carcinoma of the head and neck. Although not documented in a study population, several reports indicate a possible overlap between radiation dermatitis and the EGFRI-induced skin rash. We here present a case of severe skin reaction secondary to the addition of cetuximab to radiotherapy.

## Findings

A 56-year-old woman with squamous cell cancer of the head and neck received primary chemoradiation at our institution. Due to thrombocytopenia, chemotherapy had to be stopped early and was replaced by the EGFR inhibitor cetuximab. In a close temporal relationship to the first dose, exacerbation of a pre-existing grade 1 radiation erythema occurred within the high-dose radiation portals. Clinically, features of both the EGFRI-induced acne-like rash and radiation dermatitis coexisted within the irradiated fields. Combined EGFRI-radiotherapy was continued under close clinical surveillance without worsening of the skin reaction. The case presented here corresponds to similar experience which is increasingly published. Clinicians should be aware of the possibly severe cumulation of dermatotoxic effects in this specific therapeutic setting.

## Background

Contemporarily, there is increasing evidence supporting the concomitant use of cetuximab, a monoclonal antibody against the epidermal growth factor receptor (EGFR), in addition to high-dose radiotherapy in primary treatment concepts of head and neck cancer [1]. In comparison to conventional chemotherapy, molecularly targeted agents reveal lower haematological toxicity. However, some specific side-effects such as allergic rashes and skin reactions may limit the therapeutic use and compromise the individual patient's compliance [2].

With respect to skin reactions secondarily to the administration of EGFR inhibitors (EGFRI), pruritic follicular eruptions with either pustular or maculopapular appearance are most common with an estimated occurrence in >70% of patients [2-4]. Although acne-specific features such as comedones or microbial superinfection are lacking, the morphology is usually referred to as acneiform. Correspondingly, the clinical symptomatic is acne-like with a characteristic distribution in seborrhoeic areas (face, V-shaped neckline, upper torso). Histopathological analyses show enlargement of follicles with suppurative folliculitis and follicular plugging by keratin as consequence of the altered keratinocyte differentiation and increased apoptosis by the EGFR blockade [5].

Apparently, there is a great diversity of morphologic manifestations, and the majority of patients will experience only mild skin symptoms. However, severe reactions have been described in up to 10% of patients (grade 3/4 according to the common toxicity criteria) [3,6,7]. There is only few information available concerning possible risk factors as well as interferences with other dermatotoxic factors as, for example, concomitant radiotherapy.

## Case presentation

We report on a 56-year-old woman suffering from a squamous-cell carcinoma of the right base of tongue, 3 × 5 cm in diameter. The patient declared alcohol abuse 10 years ago, and had Child-Pugh class A liver cirrhosis. Computed tomographic (CT) scanning of the whole body revealed a stage IVA disease (cT2N2cM0). The patient was scheduled for primary hyperfractionated accelerated chemoradiation according to the German Cancer Society 95-06 schedule [8]. Radiotherapy consisted of a three-dimensional conformal technique with a concomitant boost to the primary tumour and upper neck by lateral opposed portals (72.0 Gy/45 fractions/42 days). 49.6 Gy in 29 fractions were given to the cervical lymphatics and the lower anterior neck.

In parallel to radiotherapy, systemic therapy with continuous infusional 5-fluorouracil (600 mg m^-2^, days 1–5) and mitomycin C (10 mg m^-2^, days 5 and 36) was started. However, due to progressive grade 2 thrombocytopenia, chemotherapy had to be stopped on day 4. In the progress of radiotherapy, oral grade 2 mucositis developed in week 3, and the patient got a gastrostomy placed to ensure adequate fluid and nutrition supply. To that time, the external facial and cervical skin reaction consisted of a grade 1 diffuse erythema without ulceration or epitheliolyses. The patient's regular medications were morphine sulfate and metamizole for pain relief; no other acne-inducing drugs were used.

**Figure 1:**
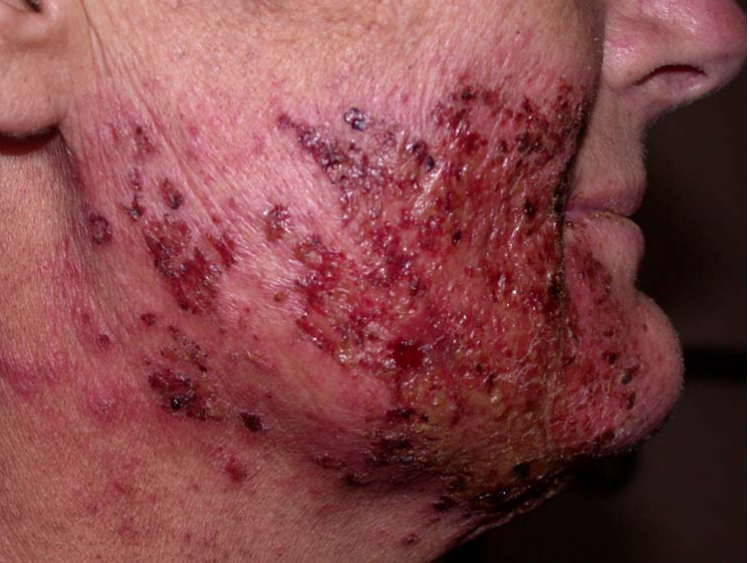
Exacerbated radiation dermatitis after cetuximab treatment.

**Figure 2:**
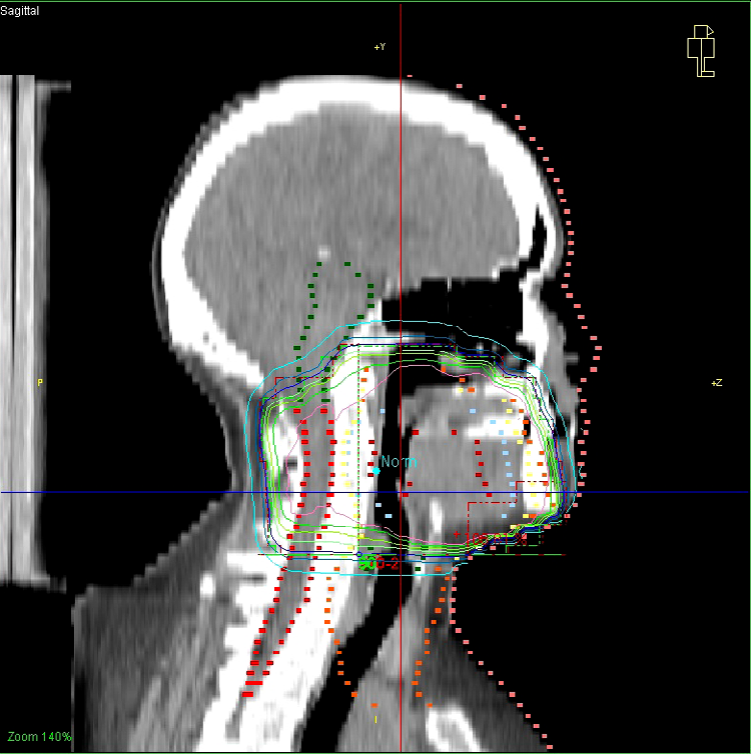
Corresponding digitally reconstructed radiograph of the radiation portals.

Due to the unforeseen stop of chemotherapy, a lack of therapeutic efficacy was assumed and, therefore, parallel treatment with cetuximab was offered according to the published protocol [1]. After informed consent, the patient received the first dose of cetuximab (250 mg m^-2^) with a radiation dose of 44.0 Gy being applied. Within hours after the first cetuximab infusion, vesicular and pustular eruptions developed on both cheeks that evolved into haemorrhagic lesions during the following days (Fig. 1). This cutaneous exacerbation was well confined to the opposed lateral oropharyngeal portals (Fig. 2). In contrast, the lower cervical skin and neckline showed only a minor erythema without ulceration or folliculitis. Hair or nail changes were not reported.

The patient got an oral antihistamine for the relief of pruritus, and a moisturising skin cream with antiseptic ingredients was applied for topical treatment. Given the stable development in the following, no other symptomatic measures were taken. Laboratory findings were normal without inflammatory changes. Herpes simplex (HSV-1) infection was ruled out by negative polymerase chain reaction findings for HSV-1 DNA, performed out of vesicular fluid as well as saliva smears. Serological tests for HSV-1 antibodies revealed positive IgG titers, but were negative for IgM.

The patient agreed to receive two further cetuximab infusions on a weekly basis. The daily visit showed no worsening of the skin reaction. At the end of treatment, the clinical findings were stable with clusters of confluent haemorrhagic crusts on both cheeks.

At follow-up six weeks after completion of therapy, the skin manifestations had declined to asymptomatic residual crusts in small areas. The patient proceeded to apply regularly moisturising emollients. Another six weeks later, intact dry skin had regenerated, but with hypopigmented stains indicating the former necrotic lesions. Repeated CT scanning at that time revealed a complete tumour response.

## Discussion

The skin rash by EGFRI is thought to be the direct consequence of the EGFR blockade in basal epidermal keratinocytes as well as the outer root sheath of hair follicles, leading to a local growth arrest and consecutive inflammation. Its occurrence may be a pharmacodynamic marker of the drug action and has been proposed as surrogate parameter of tumour response [9,10].

EGFRI are increasingly used in parallel to, or, at least, in short sequence to radiotherapy. For example, combined treatment with cetuximab and radiotherapy has been shown to improve locoregional control in patients with squamous cell carcinoma of the head and neck in a phase 3 trial [1]. Whereas the administration of cetuximab led to a significant amount of EGFRI-induced skin rashes in the combined treatment group (8.2% vs. 0.5%, p < 0.001), no statistically significant exacerbation of radiation dermatitis was reported (23 vs. 18% grade 3–5 reactions, p = 0.27).

In recent times, however, accumulating case reports reveal grade 3/4 skin reactions within radiation fields in combined treatment regimens [11-14]. The clinical localization suggests a correlation with the radiation dose as well as the former presence of intact pilosebaceous skin areas. The underlying pathomechanism remains unclear, but a synergistic inflammatory effect of both the cutaneous EGFR blockade and radiation seems likely. Accordingly, sparing of an EGFRI-induced rash has been reported in areas of soft tissue fibrosis, where previous radiotherapy has depleted skin glands and follicles [15].

The patient presented here developed ulcerative and haemorrhagic dermatitis in a close temporal relationship to the first application of cetuximab. Since we did not perform a skin biopsy, microscopic appearance and grading of this reaction remain vague. However, the clinical impression was comparable to those cases verified as grade 4 epidermal necrosis by histological analysis [11].

Most recently, first consensus guidelines for the treatment of cutaneous side-effects in combined EGFRI-radiotherapy have been published [16]. Based on the grade of the skin toxicity, treatment is adapted to the recommendations for EGFR-related rashes and radiation dermatitis. In our patient, there was a stable development of the skin reaction despite continuation of cetuximab therapy, which corresponds to reports on spontaneous resolution of skin rashes in EGFRI treatment alone [3]. However, this may be an exceptional event. In general, clinicians should be alert to the possibly severe skin toxicity after addition of EGFRI to radiotherapy. For appropriate patients, a close surveillance strategy may help to prevent further complications as well as an early treatment interruption.

## Competing interests

The author(s) declare that they have no competing interests.

## Authors' contributions

BB reviewed the patient data and drafted the manuscript, CB participated in its concept and design. Both authors read and approved the final version.
